# Managing non-SCID T cell lymphopenia after TREC-based newborn screening

**DOI:** 10.70962/jhi.20250205

**Published:** 2026-02-05

**Authors:** Annelotte J. Duintjer, Robbert G.M. Bredius, J. Merlijn van den Berg, Lisette van de Corput, Willem A. Dik, Mariëlle E. van Gijn, Stefanie S. Henriet, Evelien A. Kemper, Taco W. Kuijpers, G. Elizabeth Legger, Joris M. van Montfrans, Liesbeth H. Schölvinck, Clementien L. Vermont, Els Voorhoeve, Gert Weijman, Gijs T.J. van Well, Evelien Zonneveld-Huijssoon, Mirjam van der Burg, Maartje Blom

**Affiliations:** 1 Department of Pediatrics, Laboratory for Pediatric Immunology, Willem-Alexander Children’s Hospital, Leiden University Medical Center, Leiden, Netherlands; 2 Department of Pediatrics, Willem-Alexander Children’s Hospital, Leiden University Medical Center, Leiden, Netherlands; 3 Department of Pediatric Immunology, Rheumatology and Infectious Diseases, Emma Children’s Hospital, Amsterdam University Medical Center, Amsterdam, Netherlands; 4 Central Diagnostic Laboratory, University Medical Center Utrecht, Utrecht, Netherlands; 5 Laboratory Medical Immunology, Department of Immunology, Erasmus MC, University Medical Center, Rotterdam, Netherlands; 6 Department of Genetics, Amsterdam University Medical Center, Amsterdam, Netherlands; 7 Department of Pediatric Immunology and Infectious Diseases, Amalia Children’s Hospital, Radboud University Medical Center, Nijmegen, Netherlands; 8 Department of Clinical Chemistry, IJsselland Hospital, Capelle aan den IJssel, Netherlands; 9 Department of Pediatrics, Infectious Diseases and Immunology, Beatrix Children’s Hospital, University of Groningen, University Medical Center Groningen, Groningen, Netherlands; 10 Department of Pediatric Immunology and Infectious Diseases, Wilhelmina Children’s Hospital, University Medical Center Utrecht, Utrecht, Netherlands; 11 Department of Pediatric Immunology and Infectious Diseases, Sophia Children’s Hospital, Erasmus MC, University Medical Center, Rotterdam, Netherlands; 12 Centre for Health Protection, Dutch National Institute for Public Health and the Environment, Bilthoven, Netherlands; 13 Department for Vaccine Supply and Prevention Programs, Dutch National Institute for Public Health and the Environment, Bilthoven, Netherlands; 14 Department of Pediatrics, Division of Infectious Diseases and Immunology, MosaKids Children’s Hospital, Maastricht University Medical Center, Maastricht, Netherlands; 15 Department of Genetics, University of Groningen, University Medical Center Groningen, Groningen, Netherlands

## Abstract

Newborn screening (NBS) for severe combined immunodeficiency (SCID), based on quantifying T cell receptor excision circles (TRECs), has been increasingly implemented in screening programs worldwide. Unlike many other disorders in NBS, TREC-based screening detects many secondary findings and additional causes for T cell lymphopenia other than SCID. The clinical follow-up of SCID patients has been well implemented and documented, but newborns with other forms of T cell lymphopenia pose a new challenge for pediatric immunologists on how to offer optimal clinical management. Systematic evaluation of follow-up outcomes is crucial to establish consensus on clinical management of these patients. Here, we present clinical follow-up data of 6.5 years of NBS for SCID in the Netherlands, aiming to identify key challenges and provide recommendations. These results underscore the importance of preventing overtreatment and avoiding unnecessary prolonged follow-up and highlight the value of appropriate genetic testing and counseling. Finally, we demonstrate the critical role of international data exchange in establishing evidence-based recommendations.

## Teaser

TREC-based NBS detects many secondary findings and additional causes for T cell lymphopenia other than SCID. The clinical follow-up of these newborns poses a new challenge for pediatric immunologists. We present clinical follow-up data of 6.5 years of NBS for SCID, underscoring the importance of preventing overtreatment and avoiding unnecessary prolonged follow-up.

## Introduction

Severe combined immunodeficiency (SCID) is a group of monogenic disorders caused by absent or dysfunctional T lymphocytes and, dependent on the genetic defect, with other lymphocyte lineages often affected as well (1, 2). Patients with SCID develop early-onset severe and recurrent infections, which would be fatal within the first year of life without curative treatment in the form of allogeneic hematopoietic stem cell transplantation (HSCT) or gene therapy (3). Treatment with HSCT at a pre-symptomatic stage has been shown to be associated with improved clinical outcomes and survival (4, 5, 6, 7, 8). Because of its severity and the health gain that could be realized by early detection and initiation of preventive measures and treatment, newborn screening (NBS) for SCID has become increasingly available worldwide over the past years. The first pilot study was carried out in Wisconsin (USA) and already dates back to 2008 (9). In the Netherlands, NBS for SCID was implemented as a nationwide program on January 1, 2021, after the successful pilot study SCID-screening Onderzoek in Nederland met TRECs (SONNET), which started on April 1, 2018.

NBS for SCID is conducted by quantifying T cell receptor excision circles (TRECs) in dried blood spots (DBSs) of NBS cards, which form a marker for the maturation of T cells (10, 11). As SCID is characterized by a lack of T cells, TRECs form a sensitive biomarker for the detection of SCID (11, 12). It is known that TRECs can also be decreased in non-SCID T cell lymphopenia-associated conditions, leading to secondary findings in NBS (12, 13, 14). For example, low TRECs can be found in patients with genetic defects, other than monogenic SCID, that cause T cell impairment, such as 22q11.2 deletion syndrome and ataxia telangiectasia (15, 16, 17, 18, 19). Furthermore, TRECs can be decreased due to several reversible conditions with T cell impairment, which can include certain congenital anomalies, severe neonatal infections, and patient or maternal medication use (15, 17, 18, 20, 21, 22). Preterm birth or low birth weight can also result in low TRECs due to immature immune system development (15, 16, 17, 18, 23). Finally, TREC-based NBS identifies patients with idiopathic T cell lymphopenia, in whom no underlying clinical or genetic cause is found (15, 17, 18).

TREC-based NBS has opened the field of immunology to a new patient population of newborns with a non-SCID T cell deficiency that are being referred asymptomatically, either with an underlying genetic defect or of idiopathic origin. It is particularly notable that patients with a heterozygous *FOXN1* variant are increasingly detected by NBS. Without NBS, this patient group would not have been identified and included in immunological follow-up already from the neonatal period onward (24). Due to the introduction of TREC-based NBS, patients with idiopathic T cell lymphopenia and with heterozygous *FOXN1* variants represent a new pediatric population for which consensus, or guidelines, on clinical management from an early asymptomatic time point onward are lacking.

Determining appropriate clinical management without unnecessary interventions or excessive follow-up can therefore be challenging. Shared follow-up clinical experiences on these patients that are referred from NBS without symptoms and get included in immunological follow-up are often lacking. Therefore, a discussion arises on how to monitor these patients and to what extent to include them in follow-up. To improve NBS programs and clinical follow-up care, it is crucial to systematically collect and assess clinical outcomes of T cell impaired newborns referred from TREC-based NBS.

Here, we report the outcomes of patients identified during 6.5 years of NBS for SCID in the Netherlands. A comprehensive evaluation is provided of the diagnostic regimen and clinical follow-up of non-SCID patients with T cell lymphopenia, including patients with a heterozygous *FOXN1* variant or with idiopathic T cell lymphopenia. Based on our national clinical experiences, this observational study aimed to formulate recommendations for the clinical management of these patients while also identifying opportunities to address ongoing challenges.

## Results

### Referrals and diagnostic outcomes

During the evaluated time period of 6.5 years of TREC-based NBS, 130 newborns were referred, 62 during the pilot study (April 1, 2018–December 31, 2020) and 68 after national implementation (Table 1). In Table S1, an overview is shown of the first immunophenotyping results after referral from NBS within the different diagnostic groups. The referral rate was ∼0.03% during the pilot study (25). Between 2021 and 2024, the mean referral rate was 0.01% (26).

**Table 1.** Diagnostic outcomes of newborns referred from TREC-based NBS

​	Total referred newborns (*n* = 130)
**SCID; genotype, ** ***n*** ** (%)**	7 (5.4%)
*RAG1*	3
*IL2RG*	2
*LIG4*	1
Unknown genetic cause	1
**Non-SCID T cell impairment with genetic cause, ** ***n*** ** (%)**	38 (29.2%)
22q11.2 deletion syndrome	17
Heterozygous *FOXN1* variant	6
Trisomy 21	5
Noonan syndrome	5
Ataxia telangiectasia	2
*RMRP* variant	1
*RECQL4* variant	1
*KMT2D* variant	1
**Reversible condition with T cell impairment, ** ***n *** **(%)**	55 (42.3%)
Chylothorax and hydrops	12
(Severe) infections and sepsis	10
Maternal immunosuppressant use	9
Cardiac anomalies	6
Other^a^	18
**Preterm and/or low birth weight alone, ** ***n*** ** (%)**	6 (4.6%)
**Idiopathic T cell lymphopenia, ** ***n*** ** (%)**	12 (9.2%)
**Inconclusive** ^b^ **, ** ***n*** ** (%)**	3 (2.3%)
**Normal T cell subsets without other cause for low TRECs, ** ***n*** ** (%)**	9 (6.9%)

a Other reversible conditions included patient medication use such as corticosteroids or chemotherapy, congenital diaphragmatic hernia, asphyxia, and neonatal multimorbidity (Table S1).

b Inconclusive diagnoses included one patient that got lost to follow-up and two patients in which parents refrained from either additional diagnostics or follow-up.

Seven SCID patients were identified through NBS, one during the pilot study and six after national implementation (Table S2). Three patients were included in gene therapy trials, including two *RAG1* SCID patients treated with gene therapy in the Leiden University Medical Center (LUMC) as part of the RECOMB-project (NCT04797260) and one *IL2RG* SCID patient who received gene therapy at Great Ormond Street Hospital in London (NCT03311503 [U.S.], NCT03601286 [London]). The other four patients received HSCT, including a SCID patient with an unknown diagnosis who was treated while awaiting whole-genome sequencing (WGS) results. In parallel, an artificial thymic organoid (ATO) system was designed to rule out a thymic defect, which eventually revealed a clear early blockade in the T cell differentiation, indicating a T cell defect intrinsic to the hematopoietic stem cells of the patient (27). All SCID patients remained in good clinical condition from the moment of referral from NBS until treatment with either HSCT or gene therapy. At the time of data collection, all patients were alive and in clinically good condition.

### Non-SCID T cell impairment with genetic cause

In total, 38 referred newborns (*n* total = 130) were diagnosed with a non-SCID genetic cause for T cell impairment, including 22q11.2 deletion syndrome (*n* = 17), a heterozygous *FOXN1* variant (*n =* 6), trisomy 21 (*n =* 5), Noonan syndrome (*n* = 5), ataxia telangiectasia (*n* = 2), an *RMRP* variant (*n* = 1), a *RECQL4* variant (*n* = 1), and a *KMT2D* variant (*n* = 1). Among the 38 patients with a non-SCID genetic cause, 50% (*n* = 19) were diagnosed because of abnormal TREC results detected in NBS. This included 10 patients with 22q11.2 deletion syndrome, all heterozygous *FOXN1* patients, both ataxia telangiectasia patients, and the patient with the *RMRP* variant. In the other patients, the diagnosis was already made prenatally or shortly after birth due to dysmorphic features but independently of abnormal TREC results detected in NBS. An overview of clinical manifestations, diagnostic outcomes, and follow-up of the patients with 22q11.2 deletion syndrome (*n* = 17) is presented in Table S3.

### Heterozygous *FOXN1* patients

In six referred newborns with a diagnostically confirmed T cell lymphopenia, a heterozygous *FOXN1* variant was found (Table 2). In patient FOX-2, diagnostics also revealed leukocytosis, monocytosis, and increased B cells, eventually leading to the diagnosis of juvenile myelomonocytic leukemia (JMML). Because the clinical significance of the heterozygous *FOXN1* variant was uncertain, abnormal TRECs were thought to be the result of JMML in this patient. Immunological follow-up concerning abnormal NBS results was discontinued after 1 mo, and the patient was included in oncological follow-up. In two patients (FOX-3 and FOX-6), the same pathogenic heterozygous variant was found. These patients were treated at the same academic medical center but, as far as is known, were unrelated to each other.

**Table 2.** Overview of patients with a heterozygous *FOXN1* variant identified from TREC-based NBS

ID	Variant^a^	Referral	T cells, per µl blood (% naive)^b^	Radiological assessment of thymus	Nail dystrophy and/or skin manifestations	Infections	Prophylactic treatment	Other comorbidity	Follow-up time^c^	Follow-up ongoing
FOX-1	c.831-2A > G p.? Likely pathogenic	Non-urgent (pilot)	CD3^+^: 900 CD4^+^: 630 (41.3%) CD8^+^: 260 (92.3%)	ND	No	Viral infections, recurrent acute otitis media	Antiviral (for 3.5 mo, started 8.5 mo after referral)	No	>5 years	Yes
FOX-2	c.311T > C p.(Phe104Ser) VUS	Urgent	CD3^+^: 1,080 CD4^+^: 820 (62.8%) CD8^+^: 250 (100%)	ND	No	No	No	JMML	1 mo	No
FOX-3	c.143del p.(Cys48fs) Pathogenic	Non-urgent	CD3^+^: 1,140 CD4^+^: 770 (ND) CD8+: 310 (ND)	ND	No	No	No	No	>2 years	No
FOX-4	c.1079T > C p.(Leu360Pro) VUS	Urgent	CD3+: 1,320 CD4^+^: 1,040 (77.3%) CD8^+^: 300 (88.7%) RTE: 64%	ND	Eczema	No	No	Gastro-intestinal complaints	>1 year	No
FOX-5	c.1267del p.(Leu423Cysfs*127) Likely pathogenic	Urgent	CD3^+^: 472 CD4^+^: 444 (61.9%) CD8^+^: 27 (72%)	Small (X-thorax)	No	No	Antibacterial (for 6 mo after referral), IGRT (for 5 mo, started 1 mo after referral)	No	>1.25 years	Yes
FOX-6	c.143del p.(Cys48fs) Pathogenic	Urgent	CD3^+^: 1,050 CD4^+^: 840 (73.8%) CD8^+^: 210 (95.2%)	ND	No	No	No	No	>4 mo	Yes

IGRT, immunoglobulin replacement therapy; ND, not determined; RTE, recent thymic emigrant; VUS, variant of uncertain significance.

a Transcript: NM_001369369.1.

b First immunophenotyping results after referral from NBS.

c Refers to immunological follow-up specifically.

**Figure 1.**
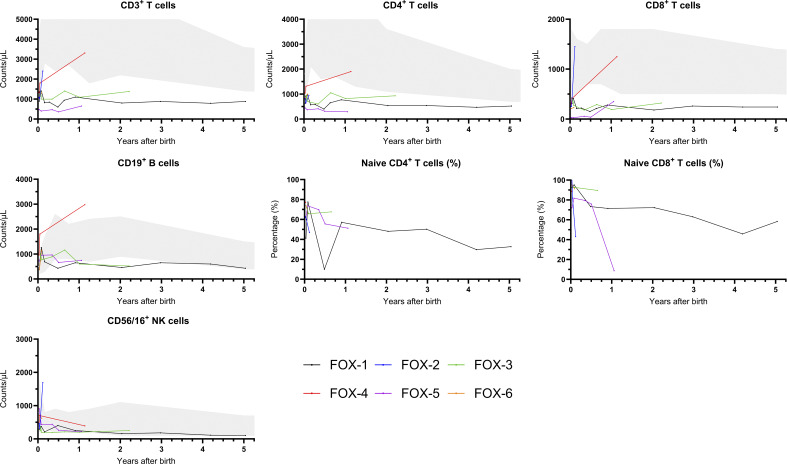
**Course of lymphocyte subsets during follow-up of patients with a heterozygous *FOXN1* variant identified from TREC-based NBS.** The grey area represents the values between the 10th and 90th percentiles.

All patients had low T cells after referral, particularly a low percentage of naive CD4^+^ T cells ranging from 41.3 to 77.3% (Fig. 1). Patient FOX-5 had the lowest T cell subsets (per µl blood; CD3^+^: 472, CD4+: 444 [61.9% naive], and CD8^+^: 27 [72% naive]), with also a notable decrease in the percentage of naive CD8^+^ T cells (8.7%) at the age of 1 year. Based on these findings, antibacterial prophylaxis was given for a duration of 6 mo (Table 2). In patient FOX-2, the JMML patient, and in patient FOX-4, an increase in T cells was observed from follow-up immunophenotyping. The other three patients (FOX-1, FOX-3, and FOX-5) showed a more stable course of low T cell counts throughout follow-up. Only patient FOX-1, who had the longest follow-up time of over 5 years, experienced recurrent infections, including viral infections and recurrent otitis media. This patient was not treated with antibacterial prophylaxis but did receive antiviral prophylaxis for a short duration of time to prevent a severe varicella zoster infection from an affected sibling. Nail dystrophy, a clinical sign that can be found in heterozygous *FOXN1* patients (24), was not described in any of the patients. Skin manifestations were present in only one patient (FOX-4), who presented with eczema. Three patients (FOX-1, FOX-5, and FOX-6) were, at the time of data collection, still in follow-up.

### Idiopathic T cell lymphopenia patients

12 referred newborns were diagnosed with idiopathic T cell lymphopenia, of which 5 during the pilot study (Table 3). In accordance with the national guideline for diagnostics after referral from NBS, whole-exome sequencing (WES) with a SCID gene panel filter was performed first in 10 patients. In the other two patients, a more extensive primary immunodeficiency (PID) gene panel filter was applied directly. In five patients, the genetic workup was not further expanded after SCID gene panel filtering. This was either because follow-up had only recently been initiated or because T cells remained stable or increased in the absence of infections. A single nucleotide polymorphism (SNP) array was performed in two patients (ITCL-7 and ITCL-9). In one patient (ITCL-7), WGS was also performed because of microcephaly and developmental delay. This revealed a heterozygous pathogenic variant in the *CSNK2A1* gene causing Okur-Chung syndrome, which was considered unlikely to explain the immunological findings from NBS. In patient ITCL-8, carriership of a pathogenic variant in the *SMARCAL1* gene was identified, but additional RNA sequencing did not reveal a second (likely) pathogenic variant.

**Table 3.** Overview of patients with idiopathic T cell lymphopenia identified from TREC-based NBS

ID	Referral	T cells, per µl blood (% naive)^a^	Genetic testing performed	Infections	Prophylactic treatment	Other comorbidity	Follow-up time^b^	Follow-up ongoing
ITCL-1	Non-urgent (pilot)	CD3^+^: 980 CD4^+^: 680 (82.1%) CD8^+^: 310 (98.3%)	WES with SCID and PID gene panel filter	Viral upper respiratory tract	Antibacterial (for 2.2 years after referral)	No	>5.5 years	Yes
ITCL-2	Non-urgent (pilot)	CD3^+^: 1,260 CD4+: 910 (ND) CD8^+^: 350 (ND)	WES with SCID gene panel filter	No	No	No	1 year	No
ITCL-3	Non-urgent (pilot)	CD3^+^: 1,020 CD4^+^: 590 (ND) CD8^+^: 450 (ND)	WES with SCID gene panel filter	No	No	No	1 year	No
ITCL-4	Non-urgent (pilot)	CD3^+^: 1160 CD4^+^: 830 (84.6%) CD8^+^: 330 (98.1%)	WES with SCID gene panel filter	No	No	No	7 mo	No
ITCL-5	Non-urgent (pilot)	CD3^+^: 1,350 CD4^+^: 780 (81.7%) CD8^+^: 530 (97.9%)	WES with SCID and PID gene panel filter	No	No	Extreme prematurity (<28 wk)	1.5 years	No
ITCL-6	Non-urgent	CD3^+^: 630 CD4^+^: 430 (70.2%) CD8^+^: 140 (67.1%)	WES with SCID gene panel filter	Viral upper respiratory tract and recurrent otitis	No	No	>3.5 years	Yes
ITCL-7	Non-urgent	CD3^+^: 1,410 CD4^+^: 1,260 (80.8%) CD8^+^: 150 (96%)	WES with SCID and PID gene panel filter, SNP array, and WGS	No	Antibacterial (for 6 mo after referral), antifungal (for 2 mo after referral)	Okur-Chung syndrome	>2.5 years	No
ITCL-8	Non-urgent	CD3^+^: 932 CD4^+^: 618 (59.2%) CD8^+^: 213 (81.7%)	WES with PID gene panel filter	No	No	No	>3.25 years	No
ITCL-9	Urgent	CD3^+^: 850 CD4^+^: 620 (80.6%) CD8^+^: 210 (63.8%)	WES with SCID and PID gene panel filter and SNP array	No	Antibacterial (started 1 month after referral, still ongoing)	No	>2 years	Yes
ITCL-10	Non-urgent	CD3^+^: 1,212 CD4^+^: 876 (59.8%) CD8^+^: 312 (94.2%)	WES with PID gene panel filter	No	No	Hiatal hernia	2 years	No
ITCL-11	Non-urgent	CD3^+^: 1,262 CD4^+^: 933 (63.6%) CD8^+^: 295 (86.1%)	WES with SCID and PID gene panel filter	No	No	No	>1.75 years	Yes
ITCL-12	Urgent	CD3^+^: 1,090 CD4^+^: 790 (74.9%) CD8^+^: 280 (83.9%)	WES with SCID gene panel filter	No	No	No	>4 mo	Yes

ND, not determined.

a First immunophenotyping results after referral from NBS.

b Refers to immunological follow-up specifically.

**Figure 2.**
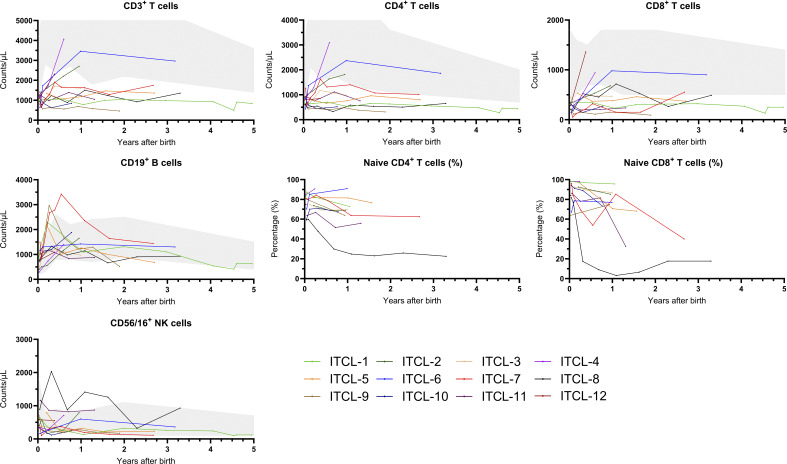
**Course of lymphocyte subsets during follow-up of patients with idiopathic T cell lymphopenia identified from TREC-based NBS.** The grey area represents the values between the 10th and 90th percentiles.

Two patients (ITCL-1 and ITCL-6) experienced recurrent infections (Table 3). Patient ITCL-1 was treated with antibacterial prophylaxis according to guidelines (trimethoprim/sulfamethoxazole) for over 2 years after referral and experienced recurrent viral upper respiratory tract infections during this period, for which the patient also visited the emergency department twice. No complications occurred, there was no need for hospitalization, and the patient recovered quickly without treatment. Patient ITCL-6 experienced recurrent viral upper respiratory tract infections and recurrent otitis in the third year of follow-up. This patient visited the emergency department once with bronchial hyperreactivity but recovered quickly and did not require hospitalization. Although this patient remained clinically stable and lymphocyte subsets improved over time, this patient remains in follow-up with yearly visits to the outpatient clinic for monitoring because of a positive family history for common variable immunodeficiency with granulomatous lymphocytic interstitial lung disease. In the other patients, no recurrent or severe infections were described. Patient ITCL-7 received antibacterial prophylaxis for 6 mo and antifungal prophylaxis for 2 mo after referral based on decreasing lymphocyte counts at second immunophenotyping (per µL blood; CD3^+^: 640, CD4^+^: 580 [80% naive], and CD8^+^: 50 [84% naive]) (Fig. 2). Prophylactic treatment was discontinued when T cells exceeded a count of 1,000 cells/μl blood. In patient ITCL-9, antibacterial prophylaxis has been continued during the entire follow-up because of persistent CD4^+^ counts of <400 cells/μl blood. In three patients (ITCL-2, ITCL-4, and ITCL-6), T cells normalized after the age of 1 year (Fig. 2). In two of the patients with persistent low T cells after one year (ITCL-7 and ITCL-8), an increase was observed toward the 10th percentile reference level at last follow-up, specifically CD3^+^ and CD8^+^ T cells, after which follow-up was ended. Five patients were at the time of data collection still in follow-up (ITCL-1, ITCL-6, ITCL-9, ITCL-11, and ITCL-12).

In five patients (ITCL-1, ITCL-5, ITCL-7, ITCL-9, and ITCL-11), administration of the live-attenuated MMR vaccination against mumps, measles, and rubella was documented and, in all, well tolerated. Three patients (ITCL-6, ITCL-8, and ITCL-9) developed a varicella zoster virus infection. In all three patients, no treatment was required and no complications occurred. Antibody response to vaccination was assessed in two patients (ITCL-6 and ITCL-7). In patient ITCL-6, there was no pneumococcal vaccine response, but there were anti-diphtheria and anti-tetanus antibodies in protective concentration present. In patient ITCL-7, sufficient protective antibodies were present against all.

## Discussion

International data on TREC-based NBS outcomes predominantly include diagnostic outcomes directly after referral and are mostly focused on clinical follow-up of patients with SCID. However, TREC-based NBS identifies more secondary findings and other causes for T cell lymphopenia than actual SCID cases (12, 13, 14). This is also supported by our study, where only 7 (5.4%) out of a total of 130 referred newborns with low TRECs (≤10 copies/3.2 mm punch, ImmunoIVD) were diagnosed with SCID during 6.5 years of NBS. Due to timely detection of SCID patients by NBS, preventive measures and prophylactic treatment were initiated promptly to prevent infections. As a result, these patients were able to undergo HSCT or participate in gene therapy trials in the most optimal clinical condition, leading to good overall outcomes after treatment. However, TREC-based NBS additionally leads to clinical and immunological follow-up of other patients with T cell lymphopenia, either of genetic or idiopathic origin. Pediatric immunologists now face complex clinical challenges regarding optimal patient management for this asymptomatic T cell lymphopenic patient population. The question arises which patients will benefit from prophylactic treatment options and which patients will be exposed to prolonged follow-up and unnecessary diagnostic testing and interventions. Routinely collected and shared long-term outcome data on these referred patients are crucial to not only better understand the natural disease course from an early stage onward but also to improve NBS programs and patient care (15, 28, 29, 30, 31).

Within the group of T cell lymphopenia patients with an underlying non-SCID genetic defect (*n* = 38), a large proportion (44.7%) consisted of patients with 22q11.2 deletion syndrome. For these and other syndromic patients, clinical guidelines or even specialized outpatient clinics exist. Challenges for clinical management mostly lie with patients carrying heterozygous *FOXN1* variants that are increasingly detected as the number of countries that have implemented TREC-based NBS has been expanding (32). Although it is known that homozygous *FOXN1* variants typically cause SCID with alopecia universalis, described as the “nude SCID phenotype,” patients with heterozygous variants can present with a generally less severe clinical phenotype with incomplete penetrance (24, 32, 33, 34). In our NBS cohort of six patients, only one patient experienced recurrent (viral) infections. In the other five patients, no recurrent or severe infections were described during the evaluated follow-up time. Moreover, no nail dystrophy or alopecia was reported in this cohort, and only one patient had eczema, which could be a skin manifestation of *FOXN1* haploinsufficiency but is highly unspecific to this condition. Therefore, in heterozygous *FOXN1* patients presenting with severe immunodeficiency suggestive of the recessive nude SCID phenotype, expanding genetic diagnostics to, if available, long-read sequencing, RNA sequencing, or WGS to find a second pathogenic variant in trans should be considered (32). In patients with a heterozygous *FOXN1* variant that show no clinical immunologic phenotype and without severe T cell lymphopenia, a watchful waiting approach without initiation of prophylaxis should be considered.

When no genetic defect or other underlying cause can be identified in referred newborns with low TRECs and T cells, the newborn is diagnosed with an idiopathic T cell lymphopenia. Patients with idiopathic T cell lymphopenia identified from TREC-based NBS can have a variable clinical presentation with either persisting or transient T cell lymphopenia (28, 35, 36, 37), which is also observed in our cohort of 12 patients. Our cohort presented with relatively mild phenotypes, with only two patients who experienced recurrent infections, predominantly viral upper respiratory tract infections. This study illustrates that consensus is lacking on follow-up regimen, as some patients with persisting T cell lymphopenia are still included in follow-up after 1 year, while others were released earlier from clinical follow-up. Similar to what is recommended for heterozygous *FOXN1* patients, also for patients with persistent idiopathic T cell lymphopenia without a clinical phenotype or severely decreased T cells, watchful waiting without prophylactic treatment should be considered. When the patient remains clinically stable, follow-up could be ended in consultation with parents. In patients with persistent severe T cell lymphopenia, for example, in some patients with ataxia telangiectasia, *FOXN1* haploinsufficiency, or 22q11.2 deletion syndrome, withholding live-attenuated vaccinations after referral from NBS should be continued until improvement to prevent serious adverse events, including vaccine-associated infections (38, 39, 40, 41).

Besides follow-up regimens, genetic diagnostics performed in this cohort also differed, as only in seven patients a PID gene panel was analyzed, in two a SNP array, and in one WGS. Expanding genetic diagnostics by performing a PID gene panel, SNP array, WES, or WGS might establish a diagnosis in some patients with persistent T cell lymphopenia. Amatuni et al. (2019) even described a diagnostic yield of 40% in patients initially diagnosed with idiopathic T cell lymphopenia, predominantly revealing DiGeorge syndrome (17). For patients with persistent T cell lymphopenia that remain in follow-up after NBS, particularly in those with an immunological or syndromal phenotype, or with a positive family history, additional genetic testing should be considered. Patients with a syndromal phenotype should be referred to a clinical geneticist to determine which genetic testing is most appropriate. In patients with only an immunological phenotype, the first step would be to perform a PID gene panel analysis.

When extending genetic diagnostics from a SCID gene panel to a broader gene panel on inborn errors of immunity (IEIs), it is crucial to provide adequate genetic counseling to parents, including information about potential incidental findings and their therapeutic and prognostic consequences. If expanding the search outside the SCID gene panel analysis, one important potential outcome to discuss with parents is the disease ataxia telangiectasia, a severe neurodegenerative disorder with a variable immunological phenotype, including low TRECs, for which no curative treatment exists (42, 43). To provide appropriate genetic counseling for parents of non-syndromic children, a stepwise genetic testing approach is applied in the Netherlands by starting with a SCID gene panel, which does not detect ataxia telangiectasia or 22q11 deletions, followed by a PID gene panel after counseling. Not all IEIs can be detected by PID gene panel analysis. For more extensive genetic testing, like WES or WGS, or more directive testing, referral to a clinical geneticist is recommended. Extending genetic diagnostics becomes increasingly important in patients with a severe idiopathic T cell lymphopenia where HSCT is under consideration, as HSCT will only be effective when there is a genetic defect affecting the hematopoiesis. Without a genetic diagnosis, the decision on HSCT will be dictated by the clinical course, and decisions about prophylactic measures and live-attenuated vaccinations are made on an individual basis. In particular, when no genetic defect is detected, a thymic model such as an ATO system could be considered as a diagnostic tool to differentiate between a thymic stromal defect and a hematopoietic intrinsic defect that could be treated with HSCT (27, 44).

For the patients with heterozygous *FOXN1* variants and idiopathic T cell lymphopenia, mild clinical phenotypes were observed in our cohort. Although we observed clinical outcomes from 6.5 years of NBS, the number of patients in these groups is small, making evidence-based recommendations for clinical management challenging. This study highlights the critical role of international data exchange in establishing recommendations based on larger cohorts. International data sharing could be established by databases with standardized data entry to evaluate long-term outcomes of patients referred from NBS. Long-term clinical follow-up data in a larger international NBS cohort will be crucial to improve clinical management and will also provide retrospective insight into which NBS findings were actionable and which were nonactionable, thereby identifying opportunities for improvement of NBS programs. Ideally, nonactionable secondary findings and false-positive referrals should be prevented as much as possible in a NBS program while maintaining high sensitivity for the target disease. Furthermore, from a societal perspective, improvements should continuously be explored to maintain public trust in screening programs, to maximize health benefits obtained from NBS, and to increase cost-effectiveness. One such improvement could be second-tier testing after TREC analysis, which would reduce these nonactionable findings and false-positive results (45, 46).

NBS using the TREC assay has been shown to be highly sensitive for detecting the target SCID, but secondary findings are found more often, including non-SCID causes with T cell impairment. Due to NBS, these newborns are detected and referred at an asymptomatic stage for which uniform guidelines on clinical care and follow-up, including potential prophylactic treatment options, are lacking. Excessive medical interventions and unnecessary prolonged follow-up should ideally be prevented. Clinical follow-up data are crucial for optimizing current referral procedures and follow-up regimens, also by exchanging knowledge and comparing data on an international level, to which international data registries are of great importance. Not only will this improve patient care, but it will also have broader implications, including improvement of the cost-effectiveness of NBS by enabling more consistent follow-up strategies and, from a parental perspective, by preventing unnecessary interventions and prolonged outpatient clinical visits. Evaluation of clinical follow-up in a systematic manner of these patients and international exchange of data can contribute to better understanding the clinical course and outcomes from an early time point onward and will help further improve management of non-SCID T cell impaired patients.

## Materials and methods

### NBS algorithm in the Netherlands

TREC-based NBS in the Netherlands is conducted with the SPOT-it Neonatal Screening kit (ImmunoIVD) using β-actin (*ACTB*) as a reference gene. During the SONNET study, a cutoff of ≤10 TREC copies/3.2 mm DBS punch was applied. When NBS for SCID was officially implemented in the Netherlands in January of 2021, the screening algorithm was adjusted to decrease false-positive referrals. Since then, newborns are either “directly” referred when TRECs are ≤2 copies/3.2 mm punch (here referred to as “urgent referral”) or a second DBS sample is requested in case of TRECs >2 and ≤10 copies/3.2 mm punch to confirm initial findings. A referral is then indicated when TRECs measured from the second DBS sample remain ≤10 copies/3.2 mm punch (here referred to as “non-urgent referral”). A second heel prick is also requested when abnormal TRECs are found in a premature newborn to measure TRECs after the gestational age of 36 wk. The complete screening algorithm has been published before (25). In case an abnormal TREC result is found, the newborn is referred to a pediatric immunologist located in one of the academic medical centers in the Netherlands within 3 days.

Following the national guideline for diagnostics after referral from NBS, as formulated by the Dutch Advisory Committee Newborn Screening for SCID, immunophenotyping results are categorized as “absent T cells” (≤200 naive CD4^+^ T cells/μl blood), “low or abnormal T cells” (≤1,500 CD3^+^ T cells/μl blood and >200 naive CD4^+^ T cells/μl blood), or “normal T cells” (>1,500 CD3^+^ T cells/μl blood and >200 naive CD4^+^ T cells/μl blood). If immunophenotyping reveals normal T cells, no further follow-up is indicated. In case T cell lymphopenia is detected, additional diagnostics are performed, which could include genetic testing. When genetics is indicated, current recommendations are to first perform WES with a SCID gene panel filter (25). If no genetic defect is found, PID gene panel filtering or other genetic diagnostics such as a SNP array can be performed if the patient has a persistent T cell lymphopenia and is suspected of having an IEI.

### Study population and data collection

All newborns that were referred from NBS to a pediatric immunologist as a result of abnormal TREC levels between the 1st of April 2018 and the 30th of September 2024 were included. Clinical data were collected at the site of all seven Dutch academic medical centers and managed using Castor’s Electronic Data Capture (v2024.2.1.0). Prior to the start of data collection, the database was validated by the designer, a data collector, and an independent third party based on the Validation Castor checklist (DS11-F01, version 5). Furthermore, data were obtained from the Dutch National Institute for Public Health and the Environment. These data included TREC results and the referral rate, which represents the percentage of newborns referred among all screened newborns during a specific time period. Data storage was pseudonymized and did not include any personal identifiers. The study was approved and declared not to apply to the Medical Research Involving Human Subjects Act by the LUMC Institutional Review Board (nr. 23-3060).

### Diagnostic categories and outcome terminology

Newborns in whom immunophenotyping was performed after referral were categorized according to the national follow-up guideline as either having absent T cells, low or abnormal T cells, or normal T cells. SCID was defined as having ≤200 naive CD4^+^ T cells/μl blood and, in general, diagnostically supported by a pathogenic genetic defect in a SCID-associated gene that conforms to the International Union of Immunological Societies classification (2). Other outcome definitions applied were in accordance with the recommendations as described by Blom et al. (2022) (46). Idiopathic T cell lymphopenia was defined as having low or abnormal T cells without an explanatory genetic or nongenetic cause.

### Statistical analysis

Descriptive statistics were performed in IBM SPSS Statistics (version 29.0.0.0 [241]). For visualization of data, GraphPad Prism (version 10.2.3 [403]) was used.

### Online supplemental material

The supplementary material includes an overview of immunophenotyping result categories within different diagnostic groups (Table S1), an overview of SCID patients identified from TREC-based NBS (Table S2), and an overview of patients with 22q11.2 deletion syndrome identified from TREC-based NBS (Table S3).

## Supplementary Material

Table S1 shows overview of immunophenotyping result categories within different diagnostic groups.

Table S2 shows overview of SCID patients identified from TREC-based NBS.

Table S3 shows overview of patients with 22q11.2 deletion syndrome identified from TREC-based NBS.

## Data Availability

The data underlying Tables 1, 2, and 3 and Figs. 1 and 2 are available in the published article and online supplemental material.

## References

[1] Fischer, A. 2000. Severe combined immunodeficiencies (SCID). Clin. Exp. Immunol. 122:143–149. 10.1046/j.1365-2249.2000.01359.x 11091267 PMC1905779

[2] Bousfiha, A.A., L. Jeddane, A. Moundir, M.C. Poli, I. Aksentijevich, C. Cunningham-Rundles, S. Hambleton, C. Klein, T. Morio, C. Picard, . 2025. The 2024 update of IUIS phenotypic classification of human inborn errors of immunity. J. Hum. Immun. 1:e20250002. 10.70962/jhi.20250002 41608113 PMC12829316

[3] Haddad, E., and M. Hoenig. 2019. Hematopoietic stem cell transplantation for severe combined immunodeficiency (SCID). Front. Pediatr. 7:481. 10.3389/fped.2019.00481 31803700 PMC6877719

[4] Heimall, J., B.R. Logan, M.J. Cowan, L.D. Notarangelo, L.M. Griffith, J.M. Puck, D.B. Kohn, M.A. Pulsipher, S. Parikh, C. Martinez, . 2017. Immune reconstitution and survival of 100 SCID patients post-hematopoietic cell transplant: A PIDTC natural history study. Blood. 130:2718–2727. 10.1182/blood-2017-05-781849 29021228 PMC5746165

[5] Lankester, A.C., B. Neven, N. Mahlaoui, E.G.J. von Asmuth, V. Courteille, M. Alligon, M.H. Albert, I.B. Serra, P. Bader, D. Balashov, . 2022. Hematopoietic cell transplantation in severe combined immunodeficiency: The SCETIDE 2006-2014 European cohort. J. Allergy Clin. Immunol. 149:1744–1754.e8. 10.1016/j.jaci.2021.10.017 34718043

[6] Thakar, M.S., B.R. Logan, J.M. Puck, E.A. Dunn, R.H. Buckley, M.J. Cowan, R.J. O’Reilly, N. Kapoor, L.F. Satter, S.-Y. Pai, . 2023. Measuring the effect of newborn screening on survival after haematopoietic cell transplantation for severe combined immunodeficiency: A 36-year longitudinal study from the primary immune deficiency treatment consortium. Lancet. 402:129–140. 10.1016/S0140-6736(23)00731-6 37352885 PMC10386791

[7] Brown, L., J. Xu-Bayford, Z. Allwood, M. Slatter, A. Cant, E.G. Davies, P. Veys, A.R. Gennery, and H.B. Gaspar. 2011. Neonatal diagnosis of severe combined immunodeficiency leads to significantly improved survival outcome: The case for newborn screening. Blood. 117:3243–3246. 10.1182/blood-2010-08-300384 21273302

[8] Pai, S.Y., B.R. Logan, L.M. Griffith, R.H. Buckley, R.E. Parrott, C.C. Dvorak, N. Kapoor, I.C. Hanson, A.H. Filipovich, S. Jyonouchi, . 2014. Transplantation outcomes for severe combined immunodeficiency, 2000-2009. N. Engl. J. Med. 371:434–446. 10.1056/NEJMoa1401177 25075835 PMC4183064

[9] Routes, J.M., W.J. Grossman, J. Verbsky, R.H. Laessig, G.L. Hoffman, C.D. Brokopp, and M.W. Baker. 2009. Statewide newborn screening for severe T-cell lymphopenia. JAMA. 302:2465–2470. 10.1001/jama.2009.1806 19996402

[10] Hazenberg, M.D., M.C. Verschuren, D. Hamann, F. Miedema, and J.J. van Dongen. 2001. T cell receptor excision circles as markers for recent thymic emigrants: Basic aspects, technical approach, and guidelines for interpretation. J. Mol. Med. 79:631–640. 10.1007/s001090100271 11715066

[11] Chan, K., and J.M. Puck. 2005. Development of population-based newborn screening for severe combined immunodeficiency. J. Allergy Clin. Immunol. 115:391–398. 10.1016/j.jaci.2004.10.012 15696101

[12] van der Spek, J., R.H. Groenwold, M. van der Burg, and J.M. van Montfrans. 2015. TREC based newborn screening for severe combined immunodeficiency disease: A systematic review. J. Clin. Immunol. 35:416–430. 10.1007/s10875-015-0152-6 25893636 PMC4438204

[13] Mauracher, A.A., F. Pagliarulo, L. Faes, S. Vavassori, T. Gungor, L.M. Bachmann, and J. Pachlopnik Schmid. 2017. Causes of low neonatal T-cell receptor excision circles: A systematic review. J. Allergy Clin. Immunol. Pract. 5:1457–1460.e22. 10.1016/j.jaip.2017.02.009 28359806

[14] Buchbinder, D., J.E. Walter, M.J. Butte, W.Y. Chan, M. Chitty Lopez, V.R. Dimitriades, M.J. Dorsey, D.J. Nugent, J.M. Puck, J. Singh, and C.A. Collins. 2021. When screening for severe combined immunodeficiency (SCID) with T cell receptor excision circles is not SCID: A case-based review. J. Clin. Immunol. 41:294–302. 10.1007/s10875-020-00931-2 33411155 PMC8179373

[15] Kwan, A., R.S. Abraham, R. Currier, A. Brower, K. Andruszewski, J.K. Abbott, M. Baker, M. Ballow, L.E. Bartoshesky, F.A. Bonilla, . 2014. Newborn screening for severe combined immunodeficiency in 11 screening programs in the United States. JAMA. 312:729–738. 10.1001/jama.2014.9132 25138334 PMC4492158

[16] Barbaro, M., A. Ohlsson, S. Borte, S. Jonsson, R.H. Zetterstrom, J. King, J. Winiarski, U. von Döbeln, and L. Hammarström. 2017. Newborn screening for severe primary immunodeficiency diseases in Sweden-a 2-year pilot TREC and KREC screening study. J. Clin. Immunol. 37:51–60. 10.1007/s10875-016-0347-5 27873105 PMC5226987

[17] Amatuni, G.S., R.J. Currier, J.A. Church, T. Bishop, E. Grimbacher, A.A. Nguyen, R. Agarwal-Hashmi, C.P. Aznar, M.J. Butte, M.J. Cowan, . 2019. Newborn screening for severe combined immunodeficiency and T-cell lymphopenia in California, 2010-2017. Pediatrics. 143:e20182300. 10.1542/peds.2018-2300 30683812 PMC6361357

[18] Argudo-Ramirez, A., A. Martin-Nalda, J.M. Gonzalez de Aledo-Castillo, R. Lopez-Galera, J.L. Marin-Soria, S. Pajares-Garcia, . 2021. Newborn screening for SCID. Experience in Spain (Catalonia). Int. J. Neonatal. Screen. 7:46. 10.3390/ijns7030046 34294672 PMC8299329

[19] Jyonouchi, S., A.M. Jongco, J. Puck, and K.E. Sullivan. 2017. Immunodeficiencies associated with abnormal newborn screening for T cell and B cell lymphopenia. J. Clin. Immunol. 37:363–374. 10.1007/s10875-017-0388-4 28353166

[20] Thomas, C., C. Monteil-Ganiere, S. Mirallie, C. Hemont, C. Dert, A. Leger, C. Joyau, D. Caldari, and M. Audrain. 2018. A severe neonatal lymphopenia associated with administration of azathioprine to the mother in a context of Crohn’s disease. J. Crohns Colitis. 12:258–261. 10.1093/ecco-jcc/jjx123 28961694

[21] Kuo, C.Y., M.I. Garcia-Lloret, P. Slev, J.F. Bohnsack, and K. Chen. 2017. Profound T-cell lymphopenia associated with prenatal exposure to purine antagonists detected by TREC newborn screening. J. Allergy Clin. Immunol. Pract. 5:198–200. 10.1016/j.jaip.2016.09.028 28065337 PMC5930011

[22] Blom, M., I. Pico-Knijnenburg, J.M. van Montfrans, R.G.M. Bredius, M. van der Burg, J.J. Swen, and D. Berghuis. 2022. Abnormal results of newborn screening for SCID after azathioprine exposure in utero: Benefit of TPMT genotyping in both mother and child. J. Clin. Immunol. 42:199–202. 10.1007/s10875-021-01138-9 34622388

[23] Atkins, A.E., M.F. Cogley, and M.W. Baker. 2021. Newborn screening for severe combined immunodeficiency: Do preterm infants require special consideration? Int. J. Neonatal. Screen. 7:40. 10.3390/ijns7030040 34287233 PMC8293075

[24] Bosticardo, M., Y. Yamazaki, J. Cowan, G. Giardino, C. Corsino, G. Scalia, R. Prencipe, M. Ruffner, D.A. Hill, I. Sakovich, . 2019. Heterozygous FOXN1 variants cause low TRECs and severe T cell lymphopenia, revealing a crucial role of FOXN1 in supporting early thymopoiesis. Am. J. Hum. Genet. 105:549–561. 10.1016/j.ajhg.2019.07.014 31447097 PMC6731368

[25] Blom, M., R.G.M. Bredius, M.E. Jansen, G. Weijman, E.A. Kemper, C.L. Vermont, I.H.I.M. Hollink, W.A. Dik, J.M. van Montfrans, M.E. van Gijn, . 2021. Parents’ perspectives and societal acceptance of implementation of newborn screening for SCID in The Netherlands. J. Clin. Immunol. 41:99–108. 10.1007/s10875-020-00886-4 33070266 PMC7846522

[26] van der Ploeg, K., O. van der Mast, and P. Verkerk. 2023. De Neonatale Hielprikscreening - Monitor 2023. TNO and Rijksinstituut voor Volksgezondheid en Milieu (RIVM). Report number: TNO 2024 R11389. https://www.pns.nl/documenten/monitor-neonatale-hielprikscreening-2023

[27] Seet, C.S., C. He, M.T. Bethune, S. Li, B. Chick, E.H. Gschweng, Y. Zhu, K. Kim, D.B. Kohn, D. Baltimore, . 2017. Generation of mature T cells from human hematopoietic stem and progenitor cells in artificial thymic organoids. Nat. Methods. 14:521–530. 10.1038/nmeth.4237 28369043 PMC5426913

[28] Vogel, B.H., V. Bonagura, G.A. Weinberg, M. Ballow, J. Isabelle, L. DiAntonio, A. Parker, A. Young, C. Cunningham-Rundles, C.-T. Fong, . 2014. Newborn screening for SCID in New York state: Experience from the first two years. J. Clin. Immunol. 34:289–303. 10.1007/s10875-014-0006-7 24578017 PMC4090801

[29] Currier, R.J. 2022. Newborn screening is on a collision course with public health ethics. Int. J. Neonatal. Screen. 8:51. 10.3390/ijns8040051 36278621 PMC9590071

[30] Speckmann, C., U. Nennstiel, M. Honig, M.H. Albert, S. Ghosh, C. Schuetz, I. Brockow, F. Hörster, T. Niehues, S. Ehl, . 2023. Prospective newborn screening for SCID in Germany: A first analysis by the pediatric immunology working group (API). J. Clin. Immunol. 43:965–978. 10.1007/s10875-023-01450-6 36843153 PMC9968632

[31] Blom, M., M. Soomann, P. Soler-Palacin, A. Sediva, A. Stray-Pedersen, R. Zetterstrom, . 2025. Newborn screening for SCID and severe T lymphocytopenia in Europe. J. Allergy Clin. Immunol. 155:377–386. 10.1016/j.jaci.2024.10.018 39510364

[32] Giardino, G., S.O. Sharapova, P. Ciznar, F. Dhalla, L. Maragliano, A. Radha Rama Devi, C. Islamoglu, A. Ikinciogullari, S. Haskologlu, F. Dogu, . 2021. Expanding the nude SCID/CID phenotype associated with FOXN1 homozygous, compound heterozygous, or heterozygous mutations. J. Clin. Immunol. 41:756–768. 10.1007/s10875-021-00967-y 33464451 PMC8068652

[33] Frank, J., C. Pignata, A.A. Panteleyev, D.M. Prowse, H. Baden, L. Weiner, L. Gaetaniello, W. Ahmad, N. Pozzi, P.B. Cserhalmi-Friedman, . 1999. Exposing the human nude phenotype. Nature. 398:473–474. 10.1038/18997 10206641

[34] Pasternak, Y., L. Vong, D. Merico, L. Abrego Fuentes, O. Scott, M. Sham, M. Fraser, A. Watts-Dickens, J. Willett Pachul, V.H.D. Kim, . 2024. Utilization of next-generation sequencing to define the role of heterozygous FOXN1 variants in immunodeficiency. J. Allergy Clin. Immunol. Glob. 3:100267. 10.1016/j.jacig.2024.100267 38800615 PMC11127205

[35] Albin-Leeds, S., J. Ochoa, H. Mehta, B.H. Vogel, M. Caggana, V. Bonagura, H. Lehman, M. Ballow, A. Rubinstein, S. Siegel, . 2017. Idiopathic T cell lymphopenia identified in New York state newborn screening. Clin. Immunol. 183:36–40. 10.1016/j.clim.2017.07.002 28694137 PMC5736366

[36] Puck, J.M. 2019. Newborn screening for severe combined immunodeficiency and T-cell lymphopenia. Immunol. Rev. 287:241–252. 10.1111/imr.12729 30565242 PMC6324582

[37] Jongco, A.M., 3rd, R. Sporter, E. Hon, O. Elshaigi, S. Zhang, F. Daian, E. Bae, A. Innamorato, C. Capo, B. Navetta-Modrov, . 2021. Characterization of infants with idiopathic transient and persistent T cell lymphopenia identified by newborn screening-a single-center experience in New York state. J. Clin. Immunol. 41:610–620. 10.1007/s10875-020-00957-6 33411154

[38] Al-Sukaiti, N., B. Reid, S. Lavi, D. Al-Zaharani, A. Atkinson, C.M. Roifman, and E. Grunebaum. 2010. Safety and efficacy of measles, mumps, and rubella vaccine in patients with DiGeorge syndrome. J. Allergy Clin. Immunol. 126:868–869. 10.1016/j.jaci.2010.07.018 20810153

[39] Rubin, L.G., M.J. Levin, P. Ljungman, E.G. Davies, R. Avery, M. Tomblyn, A. Bousvaros, S. Dhanireddy, L. Sung, H. Keyserling, . 2014. 2013 IDSA clinical practice guideline for vaccination of the immunocompromised host. Clin. Infect. Dis. 58:309–318. 10.1093/cid/cit816 24421306

[40] Buchbinder, D., F. Hauck, M.H. Albert, A. Rack, S. Bakhtiar, A. Shcherbina, E. Deripapa, K.E. Sullivan, L. Perelygina, M. Eloit, . 2019. Rubella virus-associated cutaneous granulomatous disease: A unique complication in immune-deficient patients, not limited to DNA repair disorders. J. Clin. Immunol. 39:81–89. 10.1007/s10875-018-0581-0 30607663 PMC7739844

[41] Weitering, T.J., D. Berghuis, M. Blom, M.A.A.P. Willemsen, and M. van der Burg. 2025. Clinical challenges following early detection of ataxia telangiectasia through SCID newborn screening. J. Hum. Immun. 1:e20250052. 10.70962/jhi.20250052 PMC13177395 42170582

[42] Weitering, T.J., M. Willemsen, A.M.R. Taylor, C.M.R. Weemaes, M. van der Burg, and D. Berghuis. 2023. Early diagnosis of ataxia telangiectasia through newborn screening for SCID: A case report highlighting the dilemma of pre-emptive HSCT. J. Clin. Immunol. 43:1770–1773. 10.1007/s10875-023-01571-y 37624468

[43] Blom, M., M.H.D. Schoenaker, M. Hulst, M.C. de Vries, C.M.R. Weemaes, M. Willemsen, L. Henneman, and M. van der Burg. 2019. Dilemma of reporting incidental findings in newborn screening programs for SCID: Parents’ perspective on ataxia telangiectasia. Front. Immunol. 10:2438. 10.3389/fimmu.2019.02438 31781088 PMC6851017

[44] Bosticardo, M., F. Pala, E. Calzoni, O.M. Delmonte, K. Dobbs, C.L. Gardner, N. Sacchetti, T. Kawai, E.K. Garabedian, D. Draper, . 2020. Artificial thymic organoids represent a reliable tool to study T-cell differentiation in patients with severe T-cell lymphopenia. Blood Adv. 4:2611–2616. 10.1182/bloodadvances.2020001730 32556283 PMC7322962

[45] Blom, M., I. Pico-Knijnenburg, S. Imholz, L. Vissers, J. Schulze, J. Werner, R. Bredius, and M. van der Burg. 2021. Second tier testing to reduce the number of non-actionable secondary findings and false-positive referrals in newborn screening for severe combined immunodeficiency. J. Clin. Immunol. 41:1762–1773. 10.1007/s10875-021-01107-2 34370170 PMC8604867

[46] Blom, M., R.H. Zetterstrom, A. Stray-Pedersen, K. Gilmour, A.R. Gennery, J.M. Puck, and M. van der Burg. 2022. Recommendations for uniform definitions used in newborn screening for severe combined immunodeficiency. J. Allergy Clin. Immunol. 149:1428–1436. 10.1016/j.jaci.2021.08.026 34537207 PMC9278646

