# Supplementary material for: Managing non-SCID T cell lymphopenia after TREC-based newborn screening
Source: J Hum Immun. 2026 Feb 5;2(2):e20250205. doi: 10.70962/jhi.20250205 (PMC13177657; doi:10.70962/jhi.20250205)
Supplement: Table S1 — shows overview of immunophenotyping result categories within different diagnostic groups. [file jhi_20250205_tables1.docx]

**Supplementary Material**

**Supplemental Table S1. Overview of immunophenotyping result categories within different diagnostic groups**

|  | **Absent T cells^a^,**  ***n* (%)** | **Low or abnormal T cells^b^, *n* (%)** | **Normal T cell subsets^c^,**  ***n* (%)** | **Not performed or unknown, *n (%)*** |
| --- | --- | --- | --- | --- |
| **SCID; genotype (*n* = 7)** |  |  |  |  |
| *RAG1* (*n* = 3) | 3 (100%) |  |  |  |
| *IL2RG* (*n* = 2) | 2 (100%) |  |  |  |
| *LIG4* (*n* = 1) | 1 (100%) |  |  |  |
| Unknown genetic cause (*n* = 1) | 1 (100%) |  |  |  |
| **Non-SCID T cell impairment with genetic cause (*n* = 38)** |  |  |  |  |
| 22q11.2 deletion syndrome (*n* = 17) |  | 15 (88.2%) | 2 (11.8%) |  |
| Heterozygous *FOXN1* variant (*n* = 6) |  | 6 (100%) |  |  |
| Trisomy 21 (*n* = 5) | 1 (20%) | 2 (40%) | 1 (20%) | 1 (20%) |
| Noonan syndrome (*n* = 5) | 2 (40%) |  | 3 (60%) |  |
| Ataxia telangiectasia (*n* = 2) |  | 2 (100%) |  |  |
| *RMRP* variant (*n* = 1) | 1 (100%) |  |  |  |
| *RECQL4* variant (*n* = 1) | 1 (100%) |  |  |  |
| *KMT2D* variant (*n* = 1) |  | 1 (100%) |  |  |
| **Reversible condition with T-cell impairment (*n* = 55)** |  |  |  |  |
| Chylothorax and hydrops (*n* = 12) | 7 (58.3%) | 2 (16.7%) | 1 (8.3%) | 2 (16.7%) |
| (Severe) infections and sepsis (*n* = 10) |  | 2 (20%) | 7 (70%) | 1 (10%) |
| Maternal immunosuppressant use (*n* = 9) | 2 (22.2%) | 7 (77.8%) |  |  |
| Cardiac anomalies (*n* = 6) |  | 5 (83.3%) | 1 (16.7%) |  |
| Other (*n* = 18) |  | 5 (27.8%) | 8 (44.4%) | 5 (27.8%) |
| Multimorbidity (*n* = 10)  Congenital diaphragmatic hernia (*n* = 4)  Corticosteroid treatment (*n* = 2)  Chemotherapy (*n* = 1)  Gastroschisis (*n* = 1) |  |  |  |  |
| **Preterm and/or low birth weight alone (*n* = 6)** |  | 3 (50%) | 3 (50%) |  |
| **Idiopathic T cell lymphopenia (*n* = 12)** |  | 12 (100%) |  |  |
| **Inconclusive (*n* = 3)** | 1 (33.3%) | 2 (66.7%) |  |  |
| **Normal T cell subsets without other cause for low TRECs (*n* = 9)** |  |  | 9 (100%) |  |
| **All referred patients (*n* = 130)** | 22 (16.9%) | 64 (49.2%) | 35 (26.9%) | 9 (6.9%) |

SCID = severe combined immunodeficiency; TREC = T cell receptor excision circles.

^a^ Absent T cells are defined as ≤ 200 naive CD4+ T cells/µL blood.

^b^ Low or abnormal T cells are defined as ≤ 1500 CD3+ T cells/µL blood and > 200 naive CD4+ T cells/µL blood.

^c^ Normal T cells are defined as > 1500 CD3+ T cells/µL blood and > 200 naive CD4+ T cells/µL blood.
