# Supplementary material for: Managing non-SCID T cell lymphopenia after TREC-based newborn screening
Source: J Hum Immun. 2026 Feb 5;2(2):e20250205. doi: 10.70962/jhi.20250205 (PMC13177657; doi:10.70962/jhi.20250205)
Supplement: Table S2 — shows overview of SCID patients identified from TREC-based NBS. [file jhi_20250205_tables2.docx]

**Supplemental Table S2. Overview of SCID patients identified from TREC-based NBS**

| **ID** | **Referral** | **Genotype** | **Sex** | **Consanguinuity of parents** | **T cells, per µL blood (% naive)^a^** | **Clinical manifestations** | **Treatment** |
| --- | --- | --- | --- | --- | --- | --- | --- |
| **SCID-1** | Urgent (pilot) | *IL2RG* | Male | No | CD3+: 0  CD4+ 0 (0%) | None | Antibacterial and antifungal prophylaxis, HSCT |
| **SCID-2** | Urgent | *RAG1* | Male | Yes | CD3+: 22  CD4+: 19 (ND) | None | Antibacterial and antifungal prophylaxis, IGRT, gene therapy |
| **SCID-3** | Urgent | *RAG1* | Male | Yes | CD3+: 136  CD4+: 104 (ND) | None | Antibacterial and antifungal prophylaxis, IGRT, gene therapy |
| **SCID-4** | Urgent | *RAG1* | Female | No | CD3+: 150  CD4+: 100 (ND) | None | Antibacterial and antifungal prophylaxis, IGRT, HSCT |
| **SCID-5** | Urgent | *IL2RG* | Male | No | CD3+: 10  CD4+: 0 (ND) | Varicella zoster viremia without clinical signs after passive immunization and treatment | Antibacterial, antifungal and antiviral prophylaxis, IGRT, gene therapy |
| **SCID-6** | Urgent | Unknown | Male | No | CD3+: 381  CD4+: 270 (6.7%) | None | Antibacterial and antifungal prophylaxis, IGRT, HSCT^b^ |
| **SCID-7** | Urgent | *LIG4* | Male | No | CD3+: 200  CD4+: 100 (39%) | None | Antibacterial and antifungal prophylaxis, HSCT |

ATO = artificial thymic organoid; HSCT = hematopoietic stem cell transplantation; IGRT = immunoglobulin replacement therapy; NBS = newborn screening; ND = not determined; SCID = severe combined immunodeficiency; WGS = whole genome sequencing.

^a^ First immunophenotyping results after referral from NBS.

^b^ HSCT trajectory started while awaiting WGS results, as the ATO system revealed a clear early blockade in the T cell differentiation, indicating a T cell defect intrinsic to the hematopoietic stem cells.
