# Supplementary material for: Managing non-SCID T cell lymphopenia after TREC-based newborn screening
Source: J Hum Immun. 2026 Feb 5;2(2):e20250205. doi: 10.70962/jhi.20250205 (PMC13177657; doi:10.70962/jhi.20250205)
Supplement: Table S3 — shows overview of patients with 22q11.2 deletion syndrome identified from TREC-based NBS. [file jhi_20250205_tables3.docx]

**Supplemental Table S3. Overview of patients with 22q11.2 deletion syndrome identified from TREC-based NBS**

| **ID** | **Referral** | **T cells, per µL blood (% naive)**^a^ | **Calcium** | **Radiological assessment of thymus** | **Clinical manifestations** | **Infections** | **Prophylactic treatment** | **Follow-up time^b^** | **Follow-up ongoing** |
| --- | --- | --- | --- | --- | --- | --- | --- | --- | --- |
| **DS-1** | Urgent (pilot) | CD3+: 950  CD4+: 620 (69.5%)  CD8+: 340 (98.8%) | Low | Present | Cardiac anomaly, renal tract malformations | ENT | Antibacterial (during total follow-up) | 2.8 years | No |
| **DS-2** | Urgent (pilot) | CD3+: 1660  CD4+: 1270 (82.6%)  CD8+: 380 (96.1%) | Normal | ND | Cardiac anomaly | No | No | 1.2 years | No |
| **DS-3** | Non-urgent (pilot) | CD3+: 764  CD4+: 602 (91.2%)  CD8+: 140 (91.9%) | ND | ND | Cardiac anomaly with other severe comorbidities | NA | NA | NA | Deceased |
| **DS-4** | Non-urgent (pilot) | CD3+: 1490  CD4+: 1180 (ND)  CD8+: 240 (ND) | Normal | ND | Renal tract malformations | No | No | <1 month | No |
| **DS-5** | Non-urgent | CD3+: 317  CD4+: (79.9%)  CD8+: 87 (93.4%) | ND | ND | Cardiac anomaly and renal tract malformations | No | No | 3 months | No |
| **DS-6** | Urgent | CD3+: 2163  CD4+: 1503 (81.3%)  CD8+: 625 (95.4%) | Low | Absent (ultrasound) | Unknown | Unknown | Unknown | >5 months | Unknown |
| **DS-7** | Urgent | CD3+: 1430  CD4+: 1140 (ND)  CD8+: 280 (ND) | Unknown | Unknown | Cardiac anomaly | Viral upper respiratory tract | Antibacterial (for 6 months, started 5 months after referral) | 1.5 years | No |
| **DS-8** | Non-urgent | CD3+: 1092  CD4+: 740 (27.3%)  CD8+: 347 (45.6%) | Unknown | ND | Unknown | No | No | 6 months | No |
| **DS-9** | Non-urgent | CD3+: 850  CD4+: 530 (85.8%)  CD8+: 310 (87.1%) | Normal | ND | Cardiac anomaly | No | Antibacterial (during total follow-up) and antifungal (for 2 months after referral) | 1 year | No |
| **DS-10** | Urgent | CD3+: 1200  CD4+: 730 (ND)  CD8+: 470 (ND) | ND | ND | Cardiac anomaly | No | No | 4 months | No |
| **DS-11** | Urgent | Unknown | Unknown | Unknown | Unknown | Unknown | Unknown | Unknown | Deceased |
| **DS-12** | Non-urgent | CD3+: 1000  CD4+: 720 (ND)  CD8+: 230 (ND) | Normal | ND | Cardiac anomaly | No | No | >2.5 years | Yes |
| **DS-13** | Urgent | CD3+: 689  CD4+: 599 (55.9%)  CD8+: 89 (55.8%) | Normal | Absent | Cardiac anomaly | Recurrent respiratory tract and otitis | Antibacterial (for 1.25 years after referral) and IGRT (for 4 months after referral) | >2.5 years | Yes |
| **DS-14** | Non-urgent | CD3+: 1440  CD4+: 1170 (81.3%)  CD8+: 240 (84.2%) | Normal | Inconclusive | Renal tract malformation | No | No | 1 year | No |
| **DS-15** | Non-urgent | CD3+: 1451  CD4+: 972 (75.1%)  CD8+: 463 (90.2%) | Normal | ND | Cardiac anomaly | Viral infections | No | >2 years | Yes |
| **DS-16** | Urgent | CD3+: 1256  CD4+: 1019 (ND)  CD8+: 214 (ND) | Normal | ND | Cardiac anomaly | No | No | >3 months | Yes |
| **DS-17** | Urgent | CD3+: 1148  CD4+: 679 (74.8%)  CD8+: 424 (93.5%) | Unknown | Unknown | Unknown | Early-onset neonatal sepsis | No | 1 month | No |

ENT = ear, nose, and throat; IGRT = immunoglobulin replacement therapy; NA = not applicable; NBS = newborn screening; ND = not determined; TREC = T cell receptor excision circle.

^a^ First immunophenotyping results after referral from NBS.

^b^ Refers to immunological follow-up specifically.
